# A Clinical Audit of Orthopaedic Operation Note Documentation and Digitalization of the Operative Note Template: A Quality Improvement Project

**DOI:** 10.7759/cureus.33171

**Published:** 2022-12-31

**Authors:** Omer Nasim, Abdullah Durrani, Boulos Eskander, Charalampos Pantelias, Kieran Gallagher

**Affiliations:** 1 Trauma and Orthopaedics, Poole General Hospital, Poole, GBR; 2 Trauma and Orthopaedics, University Hospitals Dorset, Poole, GBR

**Keywords:** surgical practice, clinical audit system, operative note, rcs guidlines, quality improvement research

## Abstract

Background

Good communication between a surgical team and other colleagues is vital, and the medium of communication is often the operative note. It is essential to ensure continuity of care between the operating team and other colleagues; also, it provides a medicolegal record of patient care. It checks all the four main domains of good surgical practice guidelines set by the Royal College of Surgeons (RCS) of England. The aims of this project were to evaluate the quality of operation notes against the set parameters by the RCS and to improve quality of the operative notes using information technology (IT) service software update to provide operative note digitalization.

Methods

This was a retrospective and prospective closed-loop audit, in which the operative notes were analysed for the Trauma and Orthopaedics speciality. Three separate cycles of audits were completed. In the the first cycle, data were collected retrospectively from all the operative notes, from June 1, 2020, to June 15, 2020; then, data were collected prospectively after making interventions to establish digitalization of the operative notes. The second cycle was completed from February 14 to 21, 2021, and from March 1 to 7, 2021. The third cycle was completed from August 1 to 31, 2021. All data were collected in Excel using a checklist that evaluated 34 parameters. These parameters were based on the recommendations of RCS Good Surgical Practice guidelines. All trauma and orthopaedic patients were included regardless of the type of procedure. There were no exclusion criteria in place.

Results

An overall increase from 9.5% to 66.7% in typed operative notes was achieved with the introduction of the templated operative note documentation service. There was a 40% reduction in the use of handwritten operative notes. Concerns regarding legibility were reduced in view of the digitalization of the operative notes. The first cycle of the audit, in terms of the parameters yielded, found that the operative notes were missing 10 important parameters, independent of the author grade; these were recorded in less than 10% of the operative notes. The second cycle, in terms of the parameters yielded, found that the operative notes were missing four important parameters, independent of the author grade; these were recorded in less than 10% of the operative notes. The third cycle of the audit, in terms of the parameters yielded, found that the operative notes were missing three important parameters. Specific documentation for 12 different parameters improved over the course of the three Plan-Do-Study-Act (PDSA) cycles.

Conclusion

Royal College of Surgeons guidelines and integration with IT services significantly improved the quality and legibility of operative notes that were being documented in the trauma and orthopaedics department. Structured document standards and good integration with a computer-based IT service help prompt surgeons to document in a better and easy way, thereby leading to improved clinical documentation.

## Introduction

Communication between multi-disciplinary health team members peri-operatively is essential, and the events in the operating theatre are formally documented with the help of surgical operation notes. Documentation is of paramount significance when it comes to patients' safety and their post-operative care. In keeping with good medical practice, the Royal College of Surgeons (RCS) of England has specific guidelines for operation notes, but still, in most hospitals, there is no set template software; hence, multiple variations from surgeon to surgeon, for the same procedure, are seen [1-3].

Operative notes are one of the most important medical records in a surgical unit, and maintaining a full and proper record of operative notes is the responsibility of every surgeon [3]. Anecdotally, for generations, handwritten notes in health care have had a reputation of being difficult to interpret, which can have a negative impact on patient safety, for example, incorrect surgical item counts leading to unintended retention of foreign objects in the body. Medical documentation in the Poole General Hospital is largely paper-based, and operation notes are handwritten without any set template, but on a blank page. The operative note is reliant on the author in terms of the amount of detail, adequacy, and legibility of information, and this is only available at the primary operative hospital, which makes it difficult for patient care to be easily transferable to another hospital. The general access of handwritten operation notes between hospitals (metropolitan or regional) can possibly involve contacting medical records that can potential delay patient care for hours or even days.

In legal malpractice cases, often the operative notes are presented, and studies have demonstrated that up to 45% of operative notes are indefensible from a medicolegal perspective. In the surgeons' defense, in the court, incomplete and illegible notes are often a source of weakness [2,3]. Handwritten notes are still used worldwide; however, establishing their legibility could often be a major setback [4,5].

The surgical teams and the critical care teams post-operatively analyze the operative notes as the their first-point contact with any of the formal documentation in view of understanding the patient under their review. Healthcare professionals are guided regarding the details of the procedure as well as essential post-operative care that the patient requires. Thromboprophylaxis and antibiotics are decided by the surgeons in-charge post-operatively. These critical decisions are made by surgeons on a case-to-case basis. The post-operative team often seeks guidance regarding such management in the post-operative notes. Hence, the quality of operative notes is directly related to the effective management of the surgical patient.

Therefore, in this audit we aimed to identify areas of quality improvement; we assessed the quality of operative notes against the standards set by the Royal College of Surgeons, with a view to improving the quality of operative notes and ensuring quality patient care. We also aimed to set up the digitalization of the operative notes across all surgical specialties and make a singular technological solution.

## Materials and methods

This study was conducted at the Poole General Hospital, in the surgical care group of trauma and orthopaedics. The approval for this audit was sought and granted in 2021 by the Department of Clinical Audit (reference#5319) at University Hospitals Dorset. Data in the first cycle were collected retrospectively from all the operative notes, from June 1 to 15, 2020, and then the rest were collected prospectively after making interventions in trying to establish digitalization of the operative notes. The second cycle was from February 14 to 21, 2021, and March 1 to 7, 2021. The third cycle was completed from August 1 to 31, 2021. All data were collected using a checklist that evaluated 34 parameters. The parameters used were from the Good Surgical Practice guidelines by the Royal College of Surgeons of England, and an article from the British Medical Journal [3]. All trauma and orthopaedic patients were included regardless of the type of procedure. There were no exclusion criteria in place.

The data were analyzed and presented to the surgical care group. The findings were shared with the idea of making an intervention of starting typed operative notes, with templates designed for common procedures performed routinely with editable fields for any particular specific's information required for each individual patient. Possible changes were also discussed and implemented following the meeting. A second set of data was collected, the results of which were presented in a surgical care group meeting, and subsequent changes were made to make everyone aware of the pitfalls and the progress.

Also, we audited who wrote the operation note, and whether it was written by the operating surgeon or not. Staff perceptions of communication post-typed operation note implementation were reviewed through an anonymous, opt-in, electronic survey among the surgeons and the junior doctors working on the wards, nursing staff, and allied health staff members who were involved with arranging discharges and follow-ups. Orthopaedic surgical notes and the staff perception survey were completed via a Google Forms questionnaire. A total of 50 responses were received from orthopaedic junior doctors (n=20), orthopaedic physiotherapy team (n=20), and nursing staff (n=10).

**Figure 1:**
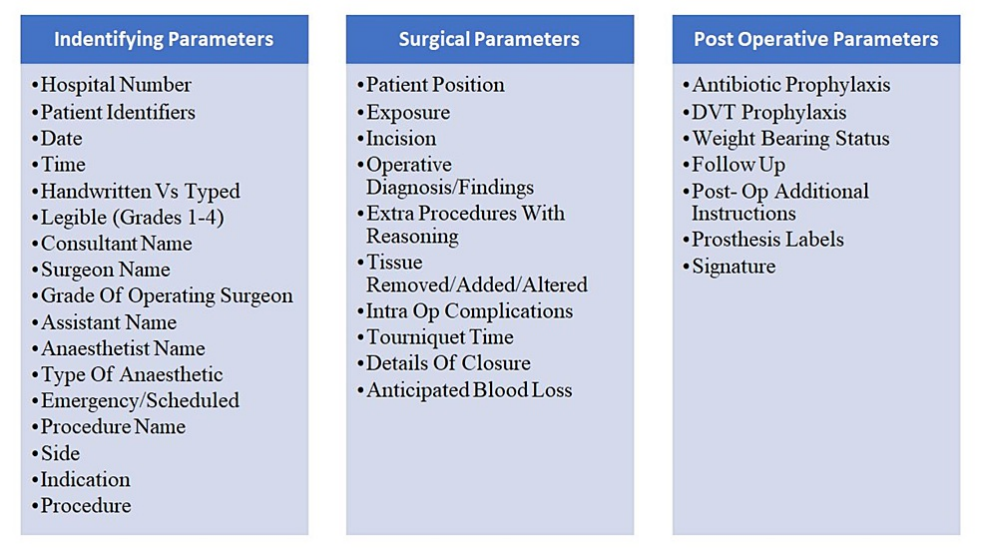
Parameters for a complete operative note as per the Royal College of Surgeons (RCS) guidelines, tabulated and grouped DVT, deep venous thrombosis Three patient identifiers include patient's full name, date of birth, address and hospital number. Legibility was graded 1-4 (the higher the score, the worse the legibility). For weight bearing status, instructions were given for post-operative mobility.

The parameters assessed for the patients were divided into three groups identifying parameters, surgical parameters, and post-operative parameters. Patient identifiers were merged into one parameter since a sticker with all the details of the patient was being used by all surgeons uniformly (Figure 1).

## Results

The first round of analysis yielded 84 operative notes; the second round of analysis yielded 96 and in the third cycle of the audit, another 84 operative notes were included. These 84 operative notes were analyzed against 34 unique criteria, which were gathered from a combination of the RCS handbook and other articles that have similar projects completed [3,4].

Legibility was graded as 1 to 4 to score the operative handwritten notes; two independent researchers evaluated them and then the scores were averaged. The higher the score, the worse was the legibility of the operative note. First cycle results, with regard to the authorship (i.e., surgeons' grade), had an interesting finding; legibility, if handwritten was worse. As anecdotally expected, the first cycle yielded more handwritten notes (76%) and then after subsequent interventions, the majority (56%) of the notes were typed by the third cycle of the audit. All cycles of the research had the same two researchers for evaluating the legibility, to minimize confounding and they were not part of the authorship or of the surgical procedure to reduce any bias.

Through the course of the audits, the percentage reduction in handwritten operative notes was reduced by 40% (Table 1). This was done partly by highlighting to the authors of the operative notes, the findings of the audit, which showed missing important components, and then also with the provision of the template typed notes, provided in each theatre; a dedicated printer was arranged as well for ease of printing the operative notes, near the recovery bay.

**Table 1:** Analysis of typed operative notes versus handwritten notes, with designated author grades SHO, senior house officer (junior doctor)

Cycles	First Cycle Findings					Second Cycle Findings					Third Cycle Findings				
Parameters	Value (n=84)	Percentage	Legibility Average (1-4)	Typed Note	Handwritten Note	Value (n=96)	Percentage	Legibility Average (1-4)	Typed Note	Handwritten Note	Value (n=84)	Percentage	Legibility Average (1-4)	Typed Note	Handwritten Note
Consultant	17	20	2	2	15	11	11	3	4	7	31	31	3	17	14
Registrar	64	76	2	6	58	73	73	2	39	34	41	41	2	31	10
SHO	3	4	1	0	3	12	12	1	5	7	12	12	2	8	4
Total	84	100%	N/A	8	76	96	100%	N/A	48	48	84	100%	N/A	56	28
Percentage	-	-	-	9.52	90.48	-	-	-	57.14	57.14	-	-	-	66.67	33.33

The first cycle of the audit, in terms of the parameters, showed that the operative notes were missing 10 important parameters, independent of the author grade; these were recorded in less than 10% of the operative notes. Whether the procedure was elective or emergency was never mentioned in the operative notes; time was not present at all in any of the operative notes (Table 2). The lack of detail in the operative notes was discernible depending on the grade; if the grade was lower, the details of the operative findings and intraoperative complications and extra procedures with reasons were not documented clearly.

**Table 2:** Analysis of the first audit cycle (with N/A adjustment), in relation to the parameters, with subdivision of operative notes by author grade SHO, senior house officer (junior doctor); DVT, deep venous thrombosis 3 Identifiers are patient identifiers that include patient's full name, date of birth, address, and hospital number. Value were adjusted with not applicable information removed, and then percentages were calculated to accurately demonstrate the completeness of the parameters (i.e., not all surgeries require a tourniquet, so that was not calculated as missing in the operative note percentages).

Parameters	Consultant	% Achieved	Registrar	% Achieved	SHO	% Achieved
Grade of surgeon	17	100	64	100	3	100
3 Identifiers	17	100	64	100	3	100
Emergency/elective	0	0.0	0	0.0	0	0.0
Date	17	100	64	100	3	100
Time	0	0.0	0	0.0	0	0.0
Consultant responsible	4	23.5	2	3.1	0	0.0
Surgeon	17	100	64	100	3	100
Assistant	15	88.2	63	98.4	2	66.7
Anaesthetist	2	11.8	2	3.1	1	33.3
Type of anaesthetic	11	64.7	52	81.3	2	66.7
Position	12	70.6	50	78.1	2	66.7
Exposure and draping	10	58.8	34	55.7	1	33.3
Procedure	16	94.1	62	96.9	3	100
Incision	16	100	51	94.4	3	100
Indication	14	82.4	59	92.2	3	100
Operative diagnosis/findings	13	76.5	47	73.4	2	66.7
Intraoperative complications	5	29.4	8	12.5	0	0.0
Extra procedures with reason	5	29.4	22	34.4	0	0.0
Tissue removed/altered	10	66.7	18	39.1	0	0.0
Prosthesis labels	11	100	40	87.0	3	100
Details of closure	13	86.7	52	100.0	3	100
Estimated blood loss	1	6.7	5	10.9	0	0.0
Tourniquet time	4	36.4	17	37.0	0	0.0
Antibiotic prophylaxis	6	35.3	16	34.8	0	0.0
DVT prophylaxis	7	41.2	30	65.2	2	66.7
Weight bearing status	12	70.6	56	87.5	3	100
Additional instructions	17	100	61	95.3	2	66.7
Follow-up	14	82.4	60	93.8	3	100
Signature	17	100	62	96.9	3	100

The second cycle of the audit, in terms of the parameters, yielded that the operative notes were missing four important parameters, independent of the author grade; these were recorded in less than 10% of the operative notes. Whether the procedure was elective or emergency was never mentioned in the operative notes; time was present in only 8% of the operative notes (Table 3). There was a marked improvement in the documentation of clear descriptions of operative details and reasons were being documented. Documentation regarding the tourniquet time was charted down in the operative note, likely because of the presentation of first audit findings and also because of prompting the surgeon to document, with the template generated in Word document.

**Table 3:** Analysis of the second audit cycle (with N/A adjustment), in relation to the parameters, with subdivision of operative notes by author grade SHO, senior house officer (junior doctor); DVT, deep venous thrombosis 3 Identifiers are patient identifiers that include patient's full name, date of birth, address, and hospital number. All values were adjusted with not applicable information removed, and then percentages were calculated, to accurately demonstrate the completeness of the parameters (i.e., not all surgeries require a tourniquet, so that was not calculated as missing in the operative note percentages).

Parameters	Consultant	% Achieved	Registrar	% Achieved	SHO	% Achieved
Grade of surgeon	11	100	73	100	12	100
3 Identifiers	11	100	73	100	12	100
Emergency/elective	0	0	0	0	0	0
Date	11	100	70	95.890411	12	100
Time	0	0	6	8.2191781	0	0
Consultant responsible	0	0	11	15.068493	1	8.333333333
Surgeon	11	100	73	100	12	100
Assistant	11	100	71	97.260274	12	100
Anaesthetist	2	18.181818	4	5.4794521	2	16.66666667
Type of anaesthetic	9	81.818182	68	93.150685	12	100
Position	9	81.818182	60	82.191781	12	100
Exposure and draping	7	63.636364	59	80.821918	10	83.33333333
Procedure	11	100	73	100	12	100
Incision	10	90.909091	70	95.890411	11	91.66666667
Indication	10	90.909091	67	91.780822	9	75
Operative diagnosis/findings	11	100	70	95.890411	12	100
Intraoperative complications	7	63.636364	39	53.424658	7	58.33333333
Extra procedures with reason	11	100	70	95.890411	11	91.66666667
Tissue removed/altered	10	90.909091	68	93.150685	12	100
Prosthesis labels	7	63.636364	44	60.273973	4	33.33333333
Details of closure	10	90.909091	65	89.041096	10	83.33333333
Estimated blood loss	1	9.0909091	10	13.69863	0	0
Tourniquet time	5	45.454545	23	31.506849	3	25
Antibiotic prophylaxis	2	18.181818	18	24.657534	6	50
DVT prophylaxis	7	63.636364	40	54.794521	5	41.66666667
Weight bearing status	8	72.727273	51	69.863014	8	66.66666667
Additional instructions	11	100	73	100	12	100
Follow-up	11	100	67	91.780822	11	91.66666667
Signature	10	90.909091	65	89.041096	12	100

The third cycle of the audit, in terms of the parameters, yielded that the operative notes were missing three important parameters, independent of the author grade; these were recorded in less than 10% of the operative notes. Whether the procedure was elective or emergency was never mentioned in the operative notes, and time was present in only 8% of the operative notes (Table 4). There was a marked improvement in the documentation of clear descriptions of operative details and reasons were being documented. Documentation regarding the tourniquet time was being charted down in the operative note, likely because of the presentation of first audit findings and also because of prompting the surgeon to document, with the template generated in Word document.

**Table 4:** Analysis of the third audit cycle (with N/A adjustment), in relation to the parameters, with subdivision of operative notes by author grade SHO, senior house officer (junior doctor); DVT, deep venous thrombosis 3 Identifiers are patient identifiers that include patient's full name, date of birth, address, and hospital number. All values were adjusted with not applicable information removed, and then percentages were calculated, to accurately demonstrate the completeness of the parameters (i.e., not all surgeries require a tourniquet, so that was not calculated as missing in the operative note percentages).

Parameters	Consultant	% Achieved	Registrar	% Achieved	SHO	% Achieved
Grade of surgeon	31	100	41	100	12	100
3 Identifiers	31	100	41	100	12	100
Emergency/elective	0	0	0	0	0	0
Date	31	100	41	100	12	100
Time	2	6.4516129	0	0	0	0
Consultant responsible	10	32.258065	5	12.195122	0	0
Surgeon	31	100	41	100	12	100
Assistant	30	96.774194	34	82.926829	12	100
Anaesthetist	9	29.032258	7	17.073171	3	25
Type of anaesthetic	21	67.741935	41	100	10	83.333333
Position	25	80.645161	40	97.560976	11	91.666667
Exposure and draping	25	80.645161	40	97.560976	11	91.666667
Procedure	31	100	41	100	12	100
Incision	28	90.322581	41	100	8	66.666667
Indication	27	87.096774	41	100	12	100
Operative diagnosis/findings	29	93.548387	41	100	12	100
Intraoperative complications	14	45.16129	26	63.414634	7	58.333333
Extra procedures with reasons	23	74.193548	35	85.365854	8	66.666667
Tissue removed/altered	29	93.548387	39	95.121951	8	66.666667
Prosthesis labels	23	74.193548	33	80.487805	6	50
Details of closure	28	90.322581	40	97.560976	10	83.333333
Estimated blood loss	2	6.4516129	5	12.195122	3	25
Tourniquet time	9	29.032258	13	31.707317	1	8.3333333
Antibiotic prophylaxis	6	19.354839	15	36.585366	1	8.3333333
DVT prophylaxis	22	70.967742	32	78.04878	9	75
Weight bearing status	27	87.096774	39	95.121951	12	100
Additional instructions	31	100	41	100	12	100
Follow-up	31	100	41	100	12	100
Signature	30	96.774194	40	97.560976	12	100

It was noticed that after the presentation of the findings in the first cycle of the audit, there was a drastic improvement in documentation with the introduction of the template operative notes, but in the third cycle, it was clear that the templates were edited out to be shorter and more generic, and the surgeons had their own particular version set up for themselves, which meant the improvement was more linear. In Table 5, it is demonstrated that the improvement in documentation from handwritten to typed notes improved drastically in all parameters under observation, but once the operative notes were mostly being typed and not handwritten, the improvement was less prominent between the second and third cycles of the audit. Also, it should be mentioned that the operative notes were not being dictated by any of the surgeons and the only record of the operative note was the one either handwritten or typed on, and was the method preferred by the operative surgeon.

**Table 5:** Sub-analysis of all three audit cycles, in relation to documentation improvement with regard to parameters DVT, deep venous thrombosis 3 Identifiers are patient identifiers that include patient's full name, date of birth, address, and hospital number. Negative values mean that there was a lack of documentation of those particular parameters in the operative note.

Parameters	First Round Analysis (%)	Difference Between Cycles (%)	Second Round Analysis (%)	Difference Between Cycles (%)	Third Round Analysis (%)
Total	100.0	0.0	100.0	0.0	100.0
3 Identifiers	100.0	0.0	100.0	0.0	100.0
Emergency/elective	0.0	0.0	0.0	0.0	0.0
Date	100.0	-3.1	96.9	3.1	100.0
Time	0.0	6.3	6.3	-3.9	2.4
Consultant responsible	7.1	5.4	12.5	5.4	17.9
Surgeon	100.0	0.0	100.0	0.0	100.0
Assistant	95.2	2.7	97.9	-7.4	90.5
Anaesthetist	6.0	2.4	8.3	14.6	22.9
Type of anaesthetic	77.4	15.3	92.7	-7.0	85.7
Position	76.2	8.2	84.4	6.1	90.5
Exposure and draping	57.1	22.9	80.0	10.5	90.5
Procedure	96.4	3.6	100.0	0.0	100.0
Incision	83.3	16.7	100.0	-2.5	97.5
Indication	90.5	-0.9	89.6	5.7	95.2
Operative diagnosis/findings	73.8	23.1	96.9	0.7	97.6
Intraoperative complications	15.5	39.7	55.2	2.8	58.0
Extra procedures with reasons	32.1	63.7	95.8	-16.3	79.5
Tissue removed/altered	33.3	65.6	98.9	-0.2	98.7
Prosthesis labels	64.3	30.5	94.8	5.2	100.0
Details of closure	81.0	14.6	95.5	3.2	98.7
Estimated blood loss	7.1	5.1	12.2	0.4	12.7
Tourniquet time	25.0	39.6	64.6	-15.6	48.9
Antibiotic prophylaxis	26.2	2.1	28.3	-0.8	27.5
DVT prophylaxis	46.4	15.5	61.9	24.4	86.3
Weight bearing status	84.5	-14.7	69.8	30.2	100.0
Additional instructions	95.2	4.8	100.0	0.0	100.0
Follow-up	91.7	1.0	92.7	7.3	100.0
Signature	97.6	-7.0	90.6	7.0	97.6

## Discussion

This audit highlights the wide variability between individuals and their level of grade and their preparation of operative notes. The study comprised solely of orthopaedic procedures, trauma (emergency) cases, and measured compliance with set standards [1,2]. Through our observations, it was evident that the transition from handwritten to typed notes would improve some areas of the parameters, as surgeons would be prompted to add information in those sections with headings present for them to fill out.

Predictably, typed operation notes increased legibility to 100%, and thus, were the preferred method for recording operation notes [4]. Other studies also found operation notes to be often below-set standards [6-9]. However, many of these studies also found that highlighting the poor standard of notes, sometimes coupled with the provision of prompts and pro formas, improved performance [6-10].

**Figure 2:**
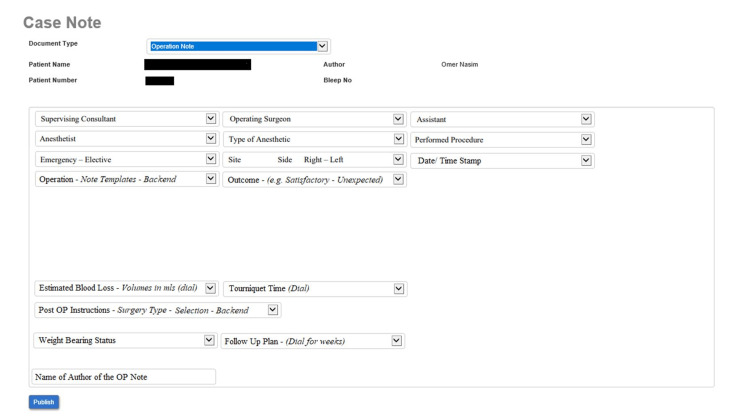
Prototype no. 1 for the IT team, designed by the audit team IT, information technology Figure is designed and made by Omer Nasim.

Positive changes were demonstrated after the first audit cycle and the changes were implemented in the second audit cycle. The improvements occurred due to the transition from handwritten to typed operative notes. The third audit cycle improved some areas of the parameters but not as drastically as the second audit cycle. This can be attributed to the fact that the comparison between the second and third cycles was between typed notes. In addition, pre-loaded data (date/time, patient details, etc.) and mandatory fields (surgical count correct, etc.) increased recorded information and the quality of content when compared to handwritten notes. Dropdown menus designed into the operative note, as seen in the information technology (IT) team prototype, would improve the use of all the parameters, as it would encourage the surgeon to fill out the sections that were earlier commonly missed, e.g. anticipated blood loss (Figure 2).

Although no formal training was provided to the authors of the operation note content, in order to improve the identified free-text areas of deficit, awareness is essential to achieve improvement. This would entail IT integration and perhaps pre-filled text for all the common routine procedures in the particular surgical group.

In this study, we used Plan-Do-Study-Act (PDSA) cycles, which include a four-step method for testing a change: by planning it, trying it, observing the results, and acting on what is learned. This is a scientific method used for action-oriented learning.

PDSA cycle 1

We did the initial audit to review the shortcomings of the operative notes, in view of the parameters selected and then presented our findings in the clinical governance meetings, to spread awareness of the lack of completion of the operative notes and how improvement with simple typed notes could be possible. We explored with the IT team whether a typed operative note could be integrated into the existing software of the hospital, but at the time, IT was not capable of managing the project. Hence, it was decided to write up and template operative notes for common procedures that were being done daily and then use those as the operative notes with editable text fields; also, a dedicated printer was provided to print these operative notes. Furthermore, other studies have shown that typed or electronic operation notes improve the quality of data recorded as compared to handwritten notes [11,12]. The discrepancy between the findings of consultants and registrars was apparent because most of the consultants preferred to do handwritten operative notes, without adding a lot of details and only writing a post-operative plan in focus, but not the findings of the procedure itself. Some of the registrars in the first cycle of the audit were still using handwritten operative notes and not following a set criteria of a template; thus, some parameters were missing.

PDSA cycle 2

**Figure 3:**
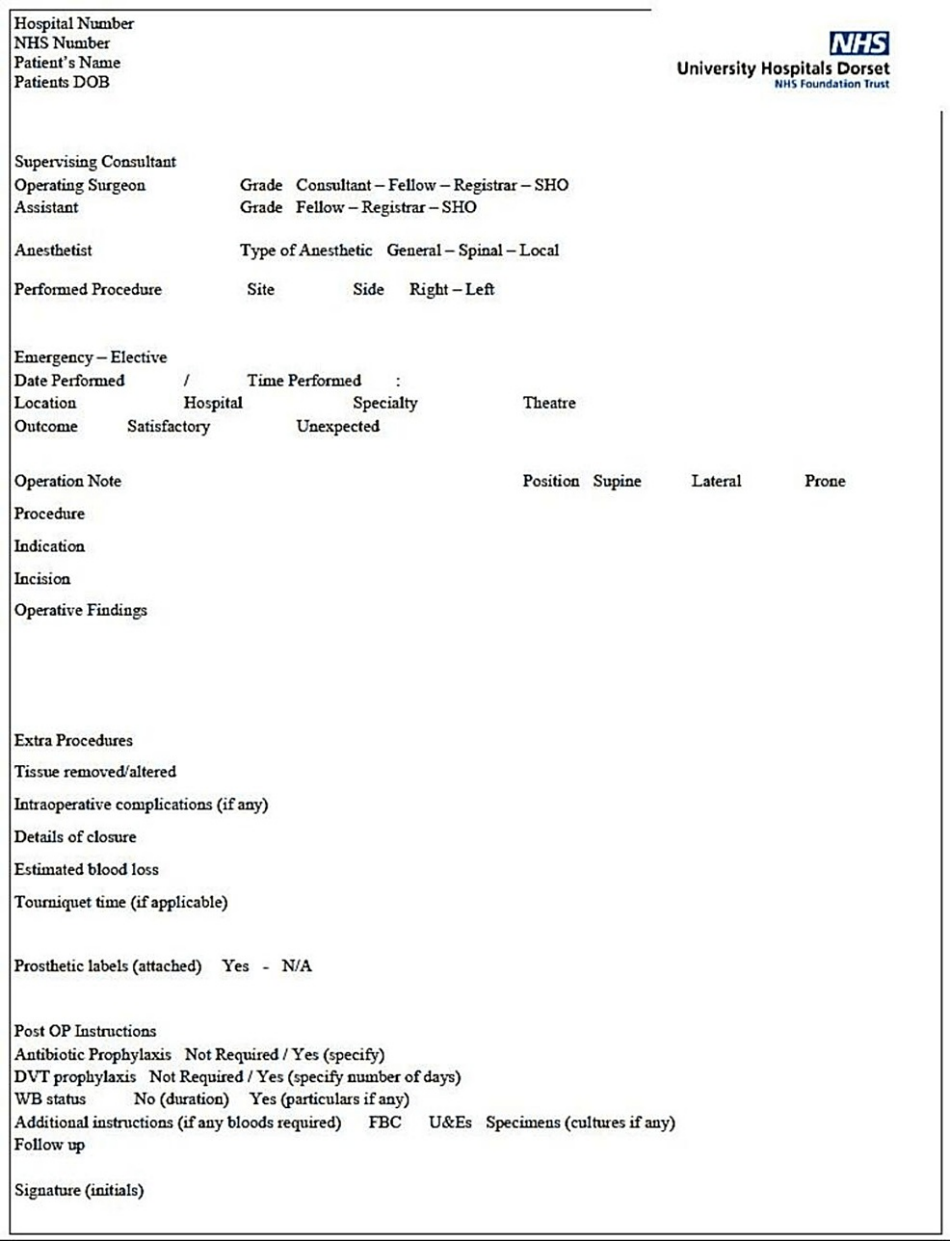
Prototype no. 2 for the IT team IT, information technology; WB, weight bearing; U&E, urea and electrolytes; FBC, full blood count; DVT, deep venous thrombosis Figure is designed and made by Omer Nasim.

The second idea was to re-present the second audit findings and demonstrate that an improvement was seen after switching from handwritten operative notes to formally typed operative notes. The opportunity to design a template operative note in the IT system was re-kindled and the IT department was keen to address this, after the demonstration of the findings of the audit, which yielded improvement in documentation with typed notes. Another template was designed for them (Figure 3). After the introduction of the templates, they were mostly adopted by the registrars and junior surgeons in the first instance, but the consultants were still more inclined towards the traditional handwritten operative notes and did not want to switch over and use the IT system operative notes. In the third cycle of the audit, it was apparent that the registrars and juniors had adopted the new template-based operative notes and the numbers of the parameters achieved were improved; there was a significant reduction in the handwritten notes.

This study shows that typed notes significantly improved the recording of intraoperative data, with findings consistent with other studies [11,12]. Therefore, switching over to electronically typed notes with parameters on the operative note templated would aid in recall to add the necessary information [13]. Furthermore, with IT integration, printed operation notes would improve the quality of note keeping; electronically stored records can be made available to medical staff to make them easily accessible for clinical use, in audit and research as well as in medicolegal procedures.

Limitations

There are some limitations to this study. As the study was conducted in a single district general hospital, the results may not be applicable to the rest of the tertiary care hospitals that have more advanced IT services. Also, this was a retrospective review, and although steps were taken to have external reviewers of the operative notes, this could have introduced selection bias. Another limitation was that the time consumption difference between the operative note being handwritten and typed was not measured in this audit, but this is something that could addressed in future audits, to see if time consumption deters the surgeon from typing the operative notes, if they do not have proficiency in typing.

## Conclusions

This quality improvement study demonstrated an improvement in the quality of operative notes when a change was made from conventional handwritten notes to typed formalized operative notes. Further work is in progress, as the hospital's quality improvement project team is trying to implement a more permanent solution. The IT team has been instructed to develop software that would make typed operative notes with dropdown menus available to be used by all surgical specialties.

## References

[1] Thomas J (2009). Medical records and issues in negligence. Indian J Urol.

[2] Breen KJ, Cordner SM, Thomson CJ, Plueckhahn VD (2010). Good Medical Practice: Professionalism, Ethics and Law. https://citeseerx.ist.psu.edu/document?repid=rep1&type=pdf&doi=4d41155f290579d10f8734034cb595156ca6cfde.

[3] Royal College of Surgeons (2022). Good Surgical Practice. https://www.rcseng.ac.uk/standards-and-research/gsp/.

[4] Yeung J (2005). Writing an operative note. BMJ.

[5] Al Hussainy H, Ali F, Jones S, McGregor-Riley JC, Sukumar S (2004). Improving the standard of operation notes in orthopaedic and trauma surgery: the value of a proforma. Injury.

[6] Payne K, Jones K, Dickenson A (2011). Improving the standard of operative notes within an oral and maxillofacial surgery department, using an operative note proforma. J Maxillofac Oral Surg.

[7] Morgan D, Fisher N, Ahmad A, Alam F (2009). Improving operation notes to meet British Orthopaedic Association guidelines. Ann R Coll Surg Engl.

[8] Bateman ND, Carney AS, Gibbin KP (1999). An audit of the quality of operation notes in an otolaryngology unit. J R Coll Surg Edinb.

[9] Mathew J, Baylis C, Saklani A, Al-Dabbagh A (2003). Quality of operative notes in a district general hospital: a time for change?. Int J Surg.

[10] McGregor-Riley J, Ali F, Al Hussainy H, Sukumar S (2003). Proformas can improve the quality of orthopaedic operation notes. J Bone Joint Surg.

[11] Atrey A, Corbett SA, Gibb PA, Singh S, Jähnich H (2010). Interactive computer operative notes for arthroscopy of joints: a free and accurate tool for surgeons. Arthroscopy.

[12] O'Bichere A, Sellu D (1997). The quality of operation notes: can simple word processors help?. Ann R Coll Surg Engl.

[13] Din R, Jena D, Muddu BN (2001). The use of an aide-memoire to improve the quality of operation notes in an orthopaedic unit. Ann R Coll Surg Engl.

